# Effects of Modified Oil Palm Kernel Expeller Fiber Enhanced via Enzymolysis Combined with Hydroxypropylation or Crosslinking on the Properties of Heat-Induced Egg White Protein Gel

**DOI:** 10.3390/molecules29225224

**Published:** 2024-11-05

**Authors:** Zhiqiang Jin, Yaoguang Gu, Wen Zhang

**Affiliations:** 1 School of Biological and Materials Engineering, Suqian University, Suqian 223800, China; guyaoguang@squ.edu.cn (Y.G.); 17169@squ.edu.cn (W.Z.); 2 Department of Biological Engineering, Suqian University, Suqian 223800, China

**Keywords:** oil palm kernel expeller dietary fiber, laccase, crosslinking, hydroxypropylation, egg white protein gel, texture quality

## Abstract

Due to its poor hydration properties, oil palm kernel expeller dietary fiber (OPKEDF) is rarely used in the food industry, especially in hydrogels, despite its advantages of high availability and low cost. To address this situation, the effects of enzymolysis combined with hydroxypropylation or crosslinking on the structure and hydration properties of OPKEDF were investigated, and the impact of these modified OPKEDFs on the properties of egg white protein gel (EWPG) was studied. Enzymolysis combined with hydroxypropylation or phosphate crosslinking improved the soluble fiber content (5.25–7.79 g/100 g), water-retention and expansion abilities of OPKEDF (*p* < 0.05). The addition of unmodified OPKEDF or modified OPKEDF increased the random coil content of EWPG and increased the density of its microstructure. Moreover, enzymolysis combined with hydroxypropylation or crosslinking enhanced the effect of OPKEDF on the properties of EWPG, including improvements in its water-retention ability, pH, hardness (from 97.96 to 195.00 g), chewiness (from 78.65 to 147.39 g), and gumminess (from 84.63 to 152.27) and a reduction in its transparency (*p* < 0.05). Additionally, OPKEDF and enzymolysis and hydroxypropylated OPKEDF increased the resilience (0.297 to 0.359), but OPKEDF treated via enzymolysis and crosslinking reduced it. Therefore, OPKEDF modified by means of enzymolysis in combination with hydroxypropylation or crosslinking improved the gel properties of EWPG. However, further work is required to determine the effects of these modifications on the nutritional profile, scalability, and economic feasibility of OPKEDF and egg white gel.

## 1. Introduction

Under the action of certain inducible factors such as heat, alkali, acid, and salt, hydrophilic polymers can interact to form a gel structure, which is a spatial network structure filled with water [1]. Hydrogels are extensively used in food, medicine, cosmetics, and other industries. Among them, protein-based hydrogels, especially egg white protein gels (EWPGs), have attracted much attention because of their unique biocompatibility, biodegradability, and easy modification [2]. EWPGs can be divided into salt-induced, alkali-induced, enzyme-induced, heat-induced, and acid-induced gels according to the particular pre-treatment methods and induction factors involved, and each of them has specific properties [3]. Heat-induced egg white gel is widely used in food, pharmaceuticals, cosmetics, and organic materials, such as steamed, boiled, and fried eggs, omelets, Japanese tofu, egg-based yogurt, preserved egg sausages, slow-release carriers for active substances, bio-dressings, nanogels, and microgels [3]. It has unique gelation properties, such as high elasticity, springiness and chewiness, but its hardness, cohesiveness, gumminess, water-retention ability, and freeze-thaw resistance are relatively lower [4]. Its high transparency is also not appropriate for application in the preservation of photosensitive food [5]. It has been shown that the gel characteristics of EWPG could be improved via the addition of polysaccharides. In prior studies, dextran sulfate increased the hardness and water-retention ability of EWPG, while a mixed gel of carboxymethyl cellulose and ovalbumin had a higher elasticity modulus [6,7,8]. Moreover, EWPGs can be endowed with the functional activities of dietary fiber (such as its hypolipidemic, hypoglycemic and antioxidation activities) via fortification. Xiao et al. [6] found that wheat bran cellulose can improve the gel properties of soy protein isolate gels. Ullah et al. [9] confirmed that okara dietary fiber can improve the gelling properties, water state, and microstructure of tofu gel. Insoluble soybean fiber can improve the emulsion gels of egg white protein [10]. 

However, to the best of our knowledge, there has been little research about the effect of dietary fiber on EWPG. Aside from its health-preserving properties, such as reducing hypertension, diabetes, obesity, gastrointestinal inflammation and cancer, dietary fiber also has extensive applications, including in gels, foaming agents, emulsifiers, antioxidants, and texture improvers [11]. The functional characteristics of dietary fiber, especially its gel properties, are positively correlated with its soluble dietary fiber (SDF) content, and dietary fiber with an SDF content of over 10 g/100 g is of high quality [12]. Cereal and oil crop byproducts are inexpensive and widely available sources of dietary fiber, but they have a low SDF content, generally less than 4 g/100 g [13,14]. Therefore, the effects of modification methods to convert insoluble dietary fiber (IDF) to SDF have been extensively studied. These modifications can change the size, microstructure, functional groups, and physicochemical properties of dietary fiber via physical, chemical, or biological methods [11,15,16,17]. Moreover, some modifications such as acid treatments, cellulase hydrolysis, hydroxypropylation and crosslinking have been proven to improve the functionality of dietary fibers, such as their hypoglycemic, hypolipidemic, antihypertensive, and antioxidant activities [11,16,18]. However, the effect of combining physical, chemical, and biological methods in the conversion of IDF to SDF has been less studied, probably because of concerns about complexity, cost, safety, and environmental pollution. Despite the potential challenges, such as complexity and concerns about effects on the environment, combining enzymatic, chemical, and physical methods is a scalable, practicable, and economical approach to improve the functional and physicochemical properties of dietary fiber.

Oil palm (*Elaeis guineensis* Jacq) expeller is a potential source of dietary fiber. The global annual yield of oil palm expeller was around 110.6 million tons in 2022 [18]. Oil palm expeller has a high fiber content (23 g∙100 g^−1^), but its soluble fiber content is only approximately 3.4 g∙100 g^−1^ [19]. Lignin and cellulose are the main components of oil palm kernel expeller dietary fiber (OPKEDF); both belong to insoluble dietary fiber, accounting for approximately 30.0 and 42.8 g∙100 g^−1^, respectively [20]. OPKEDF offers considerable adsorption capacity for sulfur dioxide and formaldehyde, but its soluble fiber content is low, and its hydration properties are poor [21,22]. Although oil palm expeller has various applications, such as animal feeds, fertilizer, acoustic absorbers, and biofuel, it is rarely used in the food industry [18,20]. Crosslinking was shown to improve the glucose sorption behavior of palm fruit bunch fiber [22]; moreover, cellulase hydrolysis has been demonstrated to reduce the insoluble fiber content and increase the solubility and functionalities such as hypoglycemic and hypolipidemic effects of dietary fibers [17,18,23]. It was also found that hydroxypropylation increased the soluble fiber content, hydration properties, and oil sorption abilities of OPKEDF [24].

Laccase is a widely investigated oxidative enzyme that can catalyze the polymerization and depolymerization of lignin. Prior studies found that it can improve some physicochemical and functional properties of dietary fibers [23,25,26]. Except for an improving effect on the hydrophilicity of fibers, phosphate crosslinking can enhance the interactions between polysaccharide chains and promote the network structure between fibers, which is advantageous for the hydration and adsorption properties of fibers [15,17]. A combination of enzymatic hydrolysis and hydroxypropylation or crosslinking may be more effective in increasing the functional properties of OPKEDF, but the relevant data are limited [17].

Therefore, OPKEDF modified by means of enzymolysis combined with hydroxypropylation (OPKEDF-EH) or phosphate crosslinking (OPKEDF-EPC) was prepared in this study. The influence of these modifications on the structure and hydration characteristics of OPKEDF and the effects of the modified OPKEDFs on the gel properties of EWPG were examined. To the best of our knowledge, this is the first investigation of the effects of composite dual enzymolysis and hydroxypropylation or crosslinking on the physicochemical properties of OPKEDF and its applications in hydrogels. This study provides new ways to improve the functional characteristics of dietary fiber and expand its applications in EWPG-based foods.

## 2. Results and Discussion

### 2.1. Effects of Different Modifications on Chemical Components of OPKEDF

In the current study, two novel and effective methods (dual enzymolysis combined with hydroxypropylation or crosslinking) were used to modify OPKEDF. The hydroxypropylation and phosphate grafting degrees of OPKEDF-EH and OPKEDF-EPC were 6.47 ± 0.22% and 1.85 ± 0.09%, respectively. The hydroxypropylation degree of OPKEDF-UEH was higher than that of OPKEDF modified via hydroxypropylation alone (3.32%) [18], demonstrating that enzymolysis increased the hydroxypropylation degree of OPKEDF. During enzymolysis, the polysaccharide chain was broken, and more chemical groups, especially free hydroxyl groups, were exposed, which can provide more grafting sites for hydroxypropyl, resulting in a higher hydroxypropylation degree [15,17]. 

As shown in Table 1, insoluble dietary fiber was the major component of the OPKEDF extracted in this study, comprising cellulose, hemicellulose, and lignin. Enzymolysis in combination with either hydroxypropylation or phosphate grafting did not show any notable influence on the protein and ash contents of OPKEDF (*p* > 0.05), but it significantly improved the SDF content (*p* < 0.05). The fat content of OPKEDF was also decreased, but the reduction was not significant (*p* > 0.05). In comparison, the IDF content of OPKEDF was obviously reduced after these modifications (*p* < 0.05), corresponding to decreased cellulose, hemicellulose, and lignin contents. Cellulase can cleave glucosidic bonds and cause the degradation of cellulose, thus reducing the cellulose content and releasing soluble monosaccharides and other hydrophilic groups [26,27]. Additionally, laccase can degrade lignin and expose its polar groups, such as phenolic acid compounds, aromatic alcohols, and other soluble fiber [28], leading to a lower lignin content. Since cellulose, hemicellulose, and lignin are all types of insoluble dietary fiber [9], cellulase hydrolysis decreased the IDF content of OPKEDF and increased the SDF content [17,25]. Apart from that, the heating and alkaline treatments during hydroxypropylation (pH 11.0, 55 °C) and crosslinking (1 mol∙L^−1^ of NaOH) can decrease hemicellulose content [15,17,27].

In addition to the above, it was observed that the introduced hydroxypropyl and phosphate groups after these modifications were responsible for the higher SDF contents of OPKEDF-EH and OPKEDF-EPC, respectively. The hydroxypropyl and phosphate groups are polar groups with high hydrophilicity [26]. OPKEDF-EH showed the highest SDF content (14.72 g∙100 g^−1^) because more non-hydrogen-bonded hydroxyl groups were exposed, and some hydroxypropyl groups were introduced into the OPKEDF after hydroxypropylation—both outcomes can increase the polarity of OPKEDF-EH [27]. Furthermore, OPKEDF-EH showed a higher SDF content than did palm kernel expeller fiber modified via hydroxypropylation alone (11.67 ± 0.25 g∙100 g^−1^) [18], evidencing that enzymolysis combined with hydroxypropylation is more effective in improving the hydrophilicity of OPKEDF than hydroxypropylation alone. The dual enzymolysis can cause the degradation of fiber chains, expose more hydrophilic groups, and thus decrease the insoluble fiber content (Table 1) [14,17]. The hydroxypropyl group has more polarity than the hydroxyl group, hydroxypropylation can increase the hydrophilicity of OPKEDF [15,24]. Therefore, a combination of enzymolysis and hydroxypropylation is more effective in improving the hydrophilicity of OPKEDF.

### 2.2. Surface Area and Color Analysis of OPKEDF

An increase in the surface area of dietary fiber can enhance its affinity with water and thus improve the textural properties of hydrogels fortified with dietary fibers [29]. After the modifications, OPKEDF showed an increased surface area and a smaller particle size (D_3,2_) (Table 1), which can probably be ascribed to the degradation of fiber chains resulting from enzymolysis [24]. Previous studies demonstrated that enzymatic hydrolysis increased the surface area of OPKEDF [17,27].

Compared with OPKEDF, OPKEDF-EH, and OPKEDF-EPC both showed a considerable color difference (*∆E*) with a decreased *L* value (representative of lightness) and an increased *a* value (indicative of redness) (*p* < 0.05), indicating that these dual modifications had a negative impact on the lightness of OPKEDF. After dual enzymolysis combined with hydroxypropylation or crosslinking, the degradation of natural pigments under heating and browning reactions occurred during the chemical modification process [30]. Kanwar et al. [15] and Zheng et al. [27] found that hydroxypropylation and enzymolysis decreased the lightness of oat dietary fiber and millet bran dietary fiber, respectively, and made their color darker.

These results in Table 1 reveal that dual enzymolysis combined with hydroxypropylation or crosslinking increased the surface area, hydrophilicity, and SDF content of OPKEDF, but reduced its lightness.

### 2.3. Structural Analysis of OPKEDF

#### 2.3.1. Surface Microstructure of OPKEDFs

**Figure 1:**
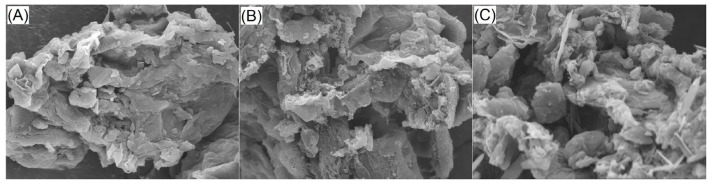
Scanning electron micrographs of OPKEDF (**A**), OPKEDF-EH (**B**), and OPKEDF-EPC (**C**) with a magnification of 5000× at 1 μm.

Scanning electron microscopy is a classic method to investigate the microstructure of samples [17]. Figure 1A–C reflect changes in the microstructure of OPKEDF after the applied modifications. OPKEDF exhibited a microstructure containing multiple cellulose tubes and debris (Figure 1A). In contrast, a distinct fragmented microstructure with greater porosity or more debris was observed in OPKEDF-EH and OPKEDF-EPC (Figure 1B,C), mainly ascribed to the breakdown of glycosidic linkages induced by dual enzymatic hydrolysis and the heating and alkali treatments applied during hydroxypropylation or crosslinking [15]. As shown in Table 1, the cellulose, lignin, and hemicellulose in OPKEDF were decreased, and the SDF content was increased after enzymolysis combined with hydroxypropylation or crosslinking; these changes revealed the degradation of fiber chains [27], resulting in a more multiple and porous microstructure. Moreover, the increased porosity and debris are predominantly attributed to the larger surface area and smaller particle size (Table 1). A larger surface area means that the chance of touching fibers and water or egg white proteins will be increased [24], which can enhance the interactions of fibers with water or egg white proteins and thus improve the textural properties of hydrogels [30].

#### 2.3.2. Fourier-Transform Infrared Spectra

**Figure 2:**
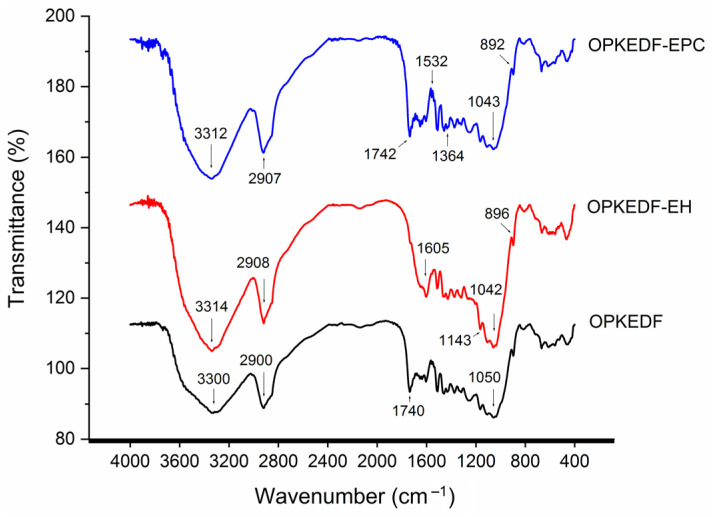
Fourier-transform infrared spectra of OPKEDF, OPKEDF-EH, and OPKEDF-EPC.

Fourier infrared spectroscopy is widely used to analyze changes in the structure of samples, especially in chemical bonds [5]. Changes in several characteristic peaks across the FT-IR spectra of OPKEDF and the modified OPKEDFs, presented in Figure 2, verified that the chemical bonds and functional groups of OPKEDF were changed by the modifications. The peak at 3300 cm^−1^ in the spectrum of OPKEDF, which represents the asymmetric stretching of O–H, transferred to 3314 and 3312 cm^−1^ in the spectra of OPKEDF-EH and OPKEDF-EPC, respectively, verifying that the hydrogen bonds in OPKEDF were changed as a result of cellulase and laccase hydrolysis combined with hydroxypropylation or crosslinking [30]. The spectra of OPKEDF-EH and OPKEDF-EPC both had a new absorption peak near 890 cm^−1^, corresponding to the vibration of β–C–H caused by cellulase hydrolysis [31]. Moreover, in the spectrum of OPKEDF-EPC, adsorption peaks at 1364 cm^−1^ (indicating the deformation of the P=O bond) and 1532 cm^−1^ (representing the vibration of C–H) confirmed successful grafting of phosphate groups on OPKEDF [12]. Additionally, in the spectrum of OPKEDF-EH, the new peak at a wavenumber of 1605 and 1143 cm^−1^ suggested that the hydroxypropyl group had been grafted onto OPKEDF [26]. In summary, the results in Figure 2 verified the successful grafting of hydroxypropyl and phosphate groups onto OPKEDF and the changes in chemical bonds and functional groups that occurred as a result of the modifications.

### 2.4. Hydration Properties of OPKEDFs

OPKEDF-EH, OPKEDF-EPC, and OPKEDF-DEA exhibited higher WRA and WEA than OPKEDF (Table 1). The improvements in the WRA and WEA of OPKEDF as a result of dual enzymolysis separately combined with hydroxypropylation and crosslinking were predominately due to (1) the greater porosity in the microstructure of OPKEDF (Figure 1) and its increased surface area (Table 1), along with (2) the grafting of hydroxypropyl and phosphate groups onto OPKEDF, enhancing its affinity with waters. Moreover, the reasons for the high WRA and WSA of OPKEDF-EPC included the network structure between the fiber chains formed after phosphate crosslinking, which could retain more water molecules [17,30]. OPKEDF-EPC exhibited a higher WEA than OPKEDF-EH (*p* < 0.05), predominately ascribed to the higher SDF content of OPKEDF-EPC. Prior studies have demonstrated that soluble fiber plays an important role in the WEA of fibers [26,31].

High viscosity is a prerequisite for polymers to form a gel [32]. The viscosities of OPKEDF-EH and OPKEDF-EPC were higher than that of OPKEDF (Table 1), in accordance with their larger surface area and higher WRA, SDF content, and WEA, as well as their more porous or multichip microstructure (Table 1 and Figure 1B,C). An improvement in the SDF content means that more fibers can contribute to the viscosity of the aqueous solution, while increases in the WRA, WEA, and surface area indicate that the interactions of fibers with waters are stronger, enhancing the viscosity of OPKEDF [29]. Furthermore, the viscosity of OPKEDF-EPC was higher than that of OPKEDF-EH (*p* < 0.05), consistent with the higher SDF content and WEA of OPKEDF-EPC, because the viscosity of a fiber was dependent on its hydrophilicity, expansion volume in aqueous solution, and mass [15].

### 2.5. Structural Characteristics of EWPGs

#### 2.5.1. Surface Microstructure of EWPGs

**Figure 3:**
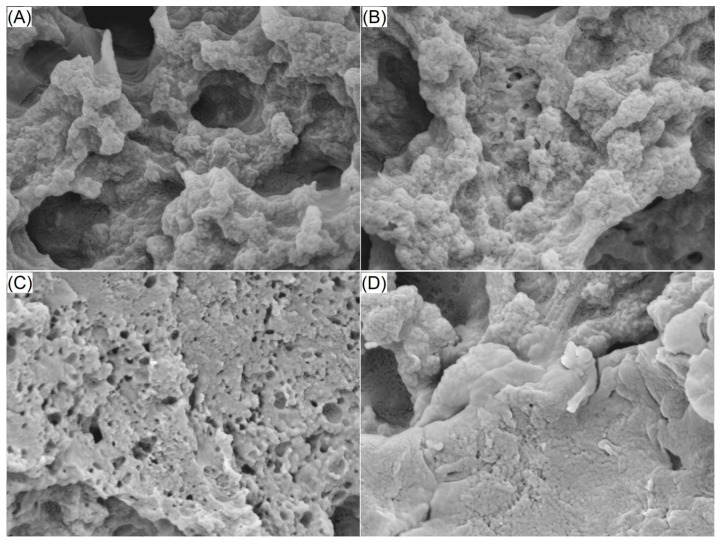
Scanning electron micrographs of native egg white gel (**A**) and egg white gels containing 5 g/100 g of OPKEDF (**B**), OPKEDF-EH (**C**), and OPKEDF-EPC (**D**) with a magnification of 30,000×, at 100 nm.

As shown in Figure 3A, the EWPG had a rough microsurface with many pores, whereas EWPG/OPKEDF, EWPG/OPKEDF-EH, and EWPG/OPKEDF-EPC all showed denser and more granular microstructures with many tiny pores (Figure 3B–D). During the preparation of heat-induced gels, the addition of fibers can provide a skeleton on which the egg white protein can aggregate, which is helpful to the formation of three-dimensional microstructures with tiny pores [10]. Additionally, the skeleton structure of the fiber matrix can enhance the interactions between egg white proteins and promote their adsorption on the surface of gels, leading to a granular microstructure [9]. In comparison with microstructure of EWPG/OPKEDF (Figure 3B), the microstructures of EWPG/OPKEDF-EPC and EWPG/OPKEDF-EH were denser with smaller tiny pores (Figure 3C,D), mainly due to their greater water expansion volume and higher viscosity (Table 1), which can improve the aggregation of egg white proteins and lead to the formation of a denser gel [31]. Moreover, phosphate groups improved the crosslinking between egg white proteins and resulted in a relatively smooth microsurface (Figure 3D) [5]. Although prior studies found that the addition of okara and star anise fibers improved the microsurface of EWPGs [9,28], to our best knowledge, the current study first demonstrated that enzymolysis combined with hydroxypropylation or crosslinking can enhance the improving effect of fibers in the microstructure of EWPG.

#### 2.5.2. Secondary Structure

The secondary structures of protein comprise α-helices, β-sheets, β-turns, and random coils, which play a crucial role in the functional properties of protein [4].

**Figure 4:**
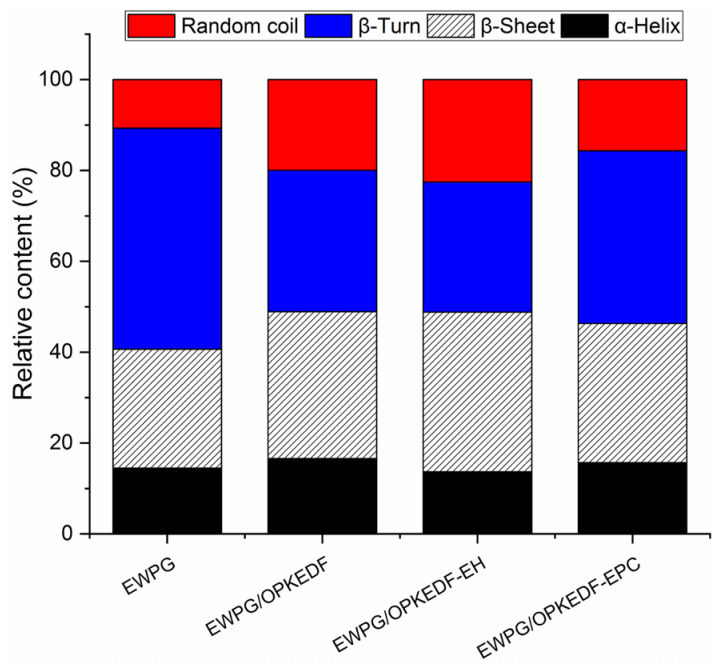
The relative content of protein secondary structure of heat-induced egg white gels fortified with OPKEDFs at an additional amount of 5 g/100 g. EWP—egg white protein gel; EWP/OPKEDF—egg white protein gel with OPKEDF; H-EWP/ OPKEDF-EH—egg white protein gel with OPKEDF-EH; H-EWP/OPKEDF-EPC—egg white gel with OPKEDF-EPC.

As Figure 4 depicts, the EWPG’s random coil and β-sheet contents were increased, and its β-turn content decreased (*p* < 0.05) correspondingly. During the formation of heat-induced gels, the structure of egg white protein is destroyed as the temperature increases; then, the stretched polypeptide chains aggregate and form a three-dimensional spatial structure through hydrogen bonds or hydrophobic forces with decreasing temperature [5]. The addition of OPKEDFs with considerable WRA and WEA (Table 1) enhanced the egg white proteins’ interactions with water and expanded their structure, leading to a denser and more granular gel, which was helpful to the formation of β-sheets and random coils [9]. The hydroxypropyl groups of OPKEDF-EH and the phosphate groups of OPKEDF-EPC were both helpful to the formation of hydrogen bonds between the egg white proteins, facilitating the formation of β-sheets in EWPG [6]. The secondary structure plays a crucial role in the gel properties of EWPG [3]. The addition of OPKEDF and the modified OPKEDFs improved the β-sheets content and microstructure of the EWPG, which is advantageous for its applications in egg-based yogurt, slow-release carriers for active substances, and microgels [5].

### 2.6. Gel Properties of EWPGs

#### 2.6.1. WRA of EWPGs

**Figure 5:**
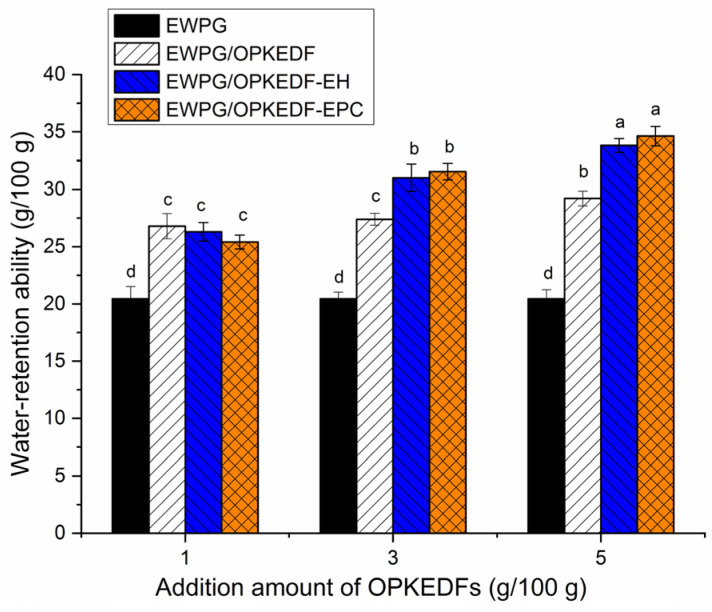
Water-retention ability of egg white protein gel and egg white protein gels fortified with OPKEDF, OPKEDF-EH, and OPKEDF-EPC. Different small letters (a–d) on the bars mean significant difference (*p* < 0.05).

Figure 5 shows that the WRA of the EWPG was increased by the addition of OPKEDF, OPKEDF-EH, and OPKEDF-EPC (*p* < 0.05), and the improvement effects were dose-dependent. One reason for this was that these OPKEDFs all have considerable WRA (1.79–3.07 g/g, Table 1), which can enhance the affinity of egg white proteins with water [7]. Another reason was that the addition of these OPKEDFs increased the random coil content of H-EWG (Figure 5) and the number of tiny pores in the microstructure (Figure 3B–D), which both assisted in the unfolding of the gel structure and the interactions between H-EWG and water molecules [9]. Furthermore, OPKEDF-EH and OPKEDF-EPC exhibited a greater ability to improve the WRA of H-EWG than did OPKEDF at addition amounts of 3–5 g/100 g, mainly attributed to their higher SDF content and WRA (Table 1) and their granular microstructures with many tiny pores (Figure 3C,D). The number of pores in the EWPG is positively related to its WRA [3,27], and a higher SDF content and WRA mean more hydrophilic fibers are available to participate in the water-retention effect of the EWPG [33]. Therefore, increases in SDF content, WRA of OPKEDF, and the number of tiny pores in the gels can improve the WRA of EWPG.

#### 2.6.2. pH Value of EWPG

**Figure 6:**
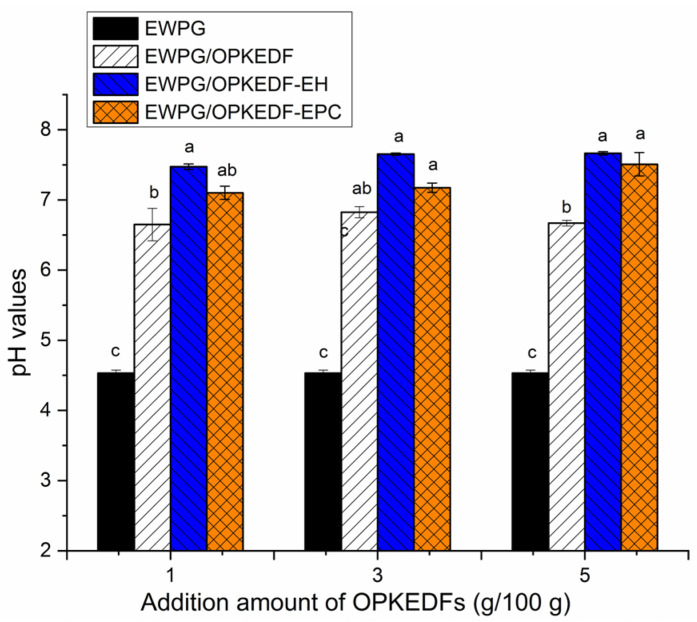
The pH values of egg white protein gel, and egg white protein gels fortified with OPKEDF, OPKEDF-EH, and OPKEDF-EPC. Different small letters (a–c) on the bars mean significant difference (*p* < 0.05).

As shown in Figure 6, the pH value of the EWPG was approximately 4.5, which is near the isoelectric point of ovalbumin (the predominant constituent of egg white proteins) [9]. Meanwhile, the pH value of the EWPG was significantly increased after the addition of OPKEDF, OPKEDF-EH and OPKEDF-EPC at 1–5 g/100 g (*p* < 0.05). At the isoelectric point, the interactions between egg white proteins are low; thus, the gels formed were weak and had low water-holding capacity and transparency [10]. However, a dense gel with a continuous network structure forms between egg white proteins at around pH 7.0 [31], which is consistent with the results shown in Figure 3B–D. Therefore, the addition of OPKEDFs increased the pH of the EWPG and thus improved its microstructure. Moreover, the pH values of EWPGs with OPKEDF were lower (*p* < 0.05) than those of EWPG with OPKEDF-EH or OPKEDF-EPC at 3–5 g/100 g, mainly because of the grafting of hydroxypropyl and acrylic groups, which can change the charge of egg white protein [26].

#### 2.6.3. Optical Transparency of EWPG

**Figure 7:**
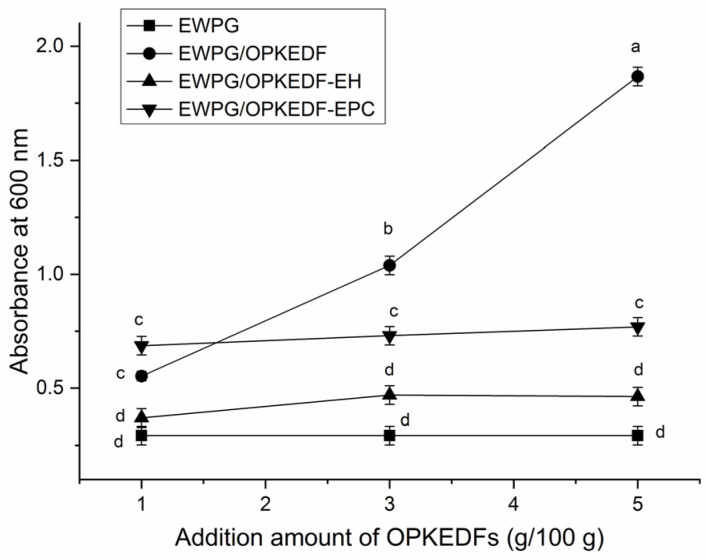
The optical transparency of egg white protein gel and egg white protein gels was fortified with OPKEDF, OPKEDF-EH, and OPKEDF-EPC. Different small letters (a–d) on the bars mean significant difference (*p* < 0.05).

A reduction in optical transparency may benefit the application of hydrogels in the preservation of photosensitive foods [5]. The effects of OPKEDFs on the optical transparency of the EWPG are shown in Figure 7.

The addition of OPKEDF, OPKEDF-EH, and OPKEDF-EPC all increased the absorbance of EWP at 600 nm and thus decreased its optical transparency (*p* < 0.05). One reason for this is that these OPKEDFs facilitated interactions between egg white proteins and allowed the formation of a denser and more granular EWP structure (Figure 3B–D), leading to greater light scattering and lower optical transparency [27]. Adding OPKEDF, OPKEDF-EH, and OPKEDF-EPC also decreased the hydrophobic force between the egg white proteins, which is helpful in decreasing the transparency of gels [33]. Furthermore, OPKEDF at 3–5 g/100 g offered the greatest reducing effect on the optical transparency of EWP, mainly ascribed to its lower WRA (Table 1) and the porous microstructure of EWPG/OPKEDF (Figure 3B). The insoluble fiber and pores in the gel can increase light scattering and reduce the gel’s transparency [10].

### 2.7. Textural Properties of EWPG

The effects of OPKEDFs on the textural properties of EWPG, including its hardness, gumminess, chewiness, cohesiveness, and resilience, are depicted in Table 2. The hardness of a gel refers to its deformation degree under external force [3]. The addition of OPKEDF, OPKEDF-EH, and OPKEDF-EPC notably enhanced the hardness of the EWPG (*p* < 0.05), mainly because OPKEDFs can provide a carbon skeleton on which the egg white protein can adsorb and aggregate [10]. Another explanation is the denser microstructures of EWPG/OPKEDFs with higher water-holding capacity (Figure 3B,D and Figure 5), which can increase a gel’s hardness [9]. The greatest hardness (195.00 g) was observed for EWPG/OPKEDF-EPC, attributed to the phosphate groups on the OPKEDF, which can facilitate protein crosslinking and increase the gel network’s strength [34].

The gumminess of the EWPG increased with increasing amounts of OPKEDFs (Table 2). Since a gel’s gumminess represents the energy needed for a semi-solid food to become stable, and it is positively correlated with its hardness, cohesiveness, and viscosity [3], it can be speculated that the increased hardness was one reason for the higher gumminess of the EWPG after the addition of OPKEDFs. The higher β-sheet content (Figure 4) can also explain the high gumminess [7]. EWPG/OPKEDF-EH showed the highest gumminess among the EWPGs, which can probably be attributed to the high viscosity and WRA of OPKEDF-EH (Table 1).

The chewiness of a gel is defined as the product of its gumminess and springiness [29]; thus, the increased gumminess was mainly responsible for the improvement in the chewiness of the EWPG after the addition of OPKEDFs. The increase in the pH (Figure 6) and water-holding capacity of the EWPG (Table 1) also facilitated the formation of a three-dimensional structure with higher chewiness [35]. OPKEDF-EPC showed the greatest enhancing effect on the chewiness of the EWPG, which was consistent with its highest viscosity and WEA (Table 1) and improvement effect on the hardness of the EWPG (Table 2). An increase in hardness, chewiness, and gumminess means more potential applications for EWPG in food, biomedicine, and cosmetics industries, such as gel beads, egg-based yogurt, double-layer emulsion, slow-release carrier for active substances, bio-dressings, nanogels, and microgels [3,36,37,38].

In contrast, the addition of OPKEDF, OPKEDF-EH and OPKEDF-EPC at 3–5 g/100 g reduced the springiness and cohesiveness of the EWPG (*p* < 0.05) (Table 2). Since the springiness of a hydrogel is positively correlated with its β-turn content [29], the decline in β-turn content of the EWPG after the addition of OPKEDFs (Figure 4) was responsible for the decreased springiness. Moreover, cohesiveness is indicative of a gel’s tensile strength. OPKEDFs increased the pH value, WRA, and hardness of the EWPG (Figure 5 and Figure 6, Table 2); these outcomes are all disadvantageous to the tight aggregation of proteins [4], resulting in lower cohesiveness. Previous studies also showed that dietary fiber can increase the strength of a gel’s network structure by providing a carbon skeleton and/or enhancing the gel’s WRA, but the springiness is reduced in consequence [10,30].

In addition, modification with OPKEDF and OPKEDF-EH at 1–5 g/100 g increased the resilience of EWPG; in contrast, OPKEDF-EPC at 1–5 g/100 g decreased the resilience of the EWPG (*p* > 0.05). The resilience of a hydrogel is dependent on its gumminess, springiness, and hardness. The enhancement effect of OPKEDF and OPKEDF-EH on the resilience of the EWPG was mainly ascribed to their enhancement effects on gumminess and hardness (Table 2). However, excessive hardness is disadvantageous to a gel’s resilience [29]; therefore, EWPG/OPKEDF-EPC showed lower resilience.

## 3. Materials and Methods

### 3.1. Materials

Craw oil palm kernel expeller harvested in September 2023 was donated by MORUO Palm Oil Processing Factory, Wenchang, China. Egg white protein powder (protein content above 95%) was purchased from Zhongyi Egg Co., Ltd., Anqing, China. Cellulase (from *Aspergillus niger*, 5.0 × 10^5^ U/g), laccase (from Trichoderma Vride G, 1.5 × 10^4^ U/g), α-amylase (from *Bacillus licheniformis*, 1.0 × 10^5^ U/g), and papain (2.5 × 10^3^ U/g) were obtained from Yizao Biochemical Co., Tianjin, China. Sodium trimetaphosphate, propylene oxide, and other reagents were analytically pure and were purchased from Dinging Reagent Co., Guangzhou, China.

### 3.2. Extraction and Cellulase Hydrolysis of OPKEDF

OPKEDF was extracted using the procedures described by Harahap et al. [22]. Briefly, craw oil palm kernel expeller was air-dried (45 °C, 3 h), milled with an LC-2TX grinder (Xinglin Mill Instrument Factory, Foshan, China), and deoiled using petroleum ether (boiling point range of 60–90 °C) in triplicate. Then, α-amylase (ratio of enzyme to defatted oil palm kernel expeller, 1:100, g/g), papain (8:1000, g/g), and glucoamylase (8:1000, g/g) were added in sequence to remove starches, proteins, and soluble carbohydrates from the defatted oil palm kernel expeller. Next, these enzymes were inactivated via heating at 100 °C for 10 min. After filtration and washing with deionized water, the residue was air-dried (52 °C, 3 h) to obtain OPKEDF.

Hydrolysis of OPKEDF by cellulase was conducted following the same procedures from Zheng et al. [27] and Xu et al. [28], respectively. Briefly, OPKEDF dispersion (1 g/15 mL, in 0.1 mol/L of acetic acid buffer, pH 4.5) was prepared, and cellulase (40 U/g) was added, which was then shaken in a BCE-002D oscillator (Shandou Thermostatic oscillator Factory, Suzhou, China) at 50 °C, and 205 rpm for 90 min [27]. Then, the pH value of the reaction dispersion was adjusted to pH 6.5 and laccase (60 U/g) was added [28]. After 60 min of shaking at 50 °C and 205 rpm, the reaction was terminated via heating (100 °C, 10 min). After cooling and filtration, the residue on the filter paper was air-dried with a GFGZ-II dryer (Shaanxi Core Dry Factory, Hanzhong, China) (45 °C, 6 h) to obtain cellulase hydrolyzed oil palm kernel expeller dietary fiber (OPKEDF-E).

### 3.3. Hydroxypropylation of OPKEDF-E

OPKEDF-E (7.5 g) was dispersed in 150 mL of dH_2_O and shaken in a BCE-002D oscillator (Shangyu Jingke Instrument Factory, Hangzhou, China) at 195 r∙min^−1^ and 55 °C [17]. Five minutes later, the dispersion was adjusted to pH 11.0, and Na_2_SO_4_ was slowly added until the final concentration was 20 mg∙mL^−1^. Next, propylene oxide (3 mL) was added, and the dispersion was shaken in the EZC-004H oscillator (Zhuji Food Instrument Factory, Shaoxing, China) at 195 r∙min^−1^ and 40 °C. Twenty-four hours later, the reaction mixture was filtered. The residue was dehydrated with the GFGZ-II dryer (Shaanxi Core Dry Factory, Hanzhong, China) (50 °C, 6 h), and OPKEDF modified by means of enzymolysis and hydroxypropylation (OPKEDF-EH) was thus obtained. The hydroxypropyl degree was measured using the procedures described by Torres-Pérez et al. [24].

### 3.4. Crosslinking of OPKEDF-E

OPKEDF-E (20 g), sodium tripolyphosphate (0.5 g) and sodium trimetaphosphate (5 g) were dispersed in 200 mL deionized water (dH_2_O); the mixture was adjusted to pH 11.0 with 1 mol∙L^−1^ of NaOH [20]. The reaction was stirred at 300 rpm for 180 min at 45 °C and then stopped by adjusting the pH to 7.0 using 1 mol∙L^−1^ of HCl. After filtration, the residue was collected and washed using dH_2_O (80 mL). The washed residue was dried at 40 °C overnight to obtain OPKEDF modified by enzymatic hydrolysis and phosphate crosslinking (OPKEDF-EPC).

### 3.5. Preparation of Egg White Protein OPKEDF Gel

Fifteen grams of egg white powder was dissolved in 100 mL dH_2_O and then kept at 4 °C overnight. A volume of 20 mL of egg white solution was transferred into a 50 mL beaker, and then an amount (1, 3, and 5 g/100 g) of OPKEDF, OPKEDF-EH, and OPKEDF-EPC was added. The mixture in the beaker was gently stirred in the BHE-002Z shaker at 90 °C for 30 min. This process included two stages: stirring for 3 min at 160 rpm and holding for 27 min at 0 rpm [17]. Finally, the mixtures were cooled under running tap water and kept at 4 °C for 7 h to get unmodified egg white protein gel (EWPG) and egg white protein gels separately fortified with OPKEDF (EWPG/OPKEDF), OPKEDF-EH (EWPG/OPKEDF-EH), or OPKEDF-EPC (EWPG/OPKEDF-EPC).

### 3.6. Chemical Constituents, Surface Area, and Color Determinations

The soluble, insoluble, and total dietary fiber contents of OPKEDF, OPKEDF-EH, and OPKEDF-EPC were determined using the AOAC.991.43 method [39]. The methods AOAC.955.04, AOAC.920.39, AOAC.924.05 and AOAC.92.05 were employed to determine the ash, protein, moisture, and fat contents of the OPKEDFs, respectively [39]. The surface area (m^2^∙kg^−1^) and particle size (Sauter mean diameter, D_3,2_) were analyzed with a JFNER-LB particle size analyzer (Jingbei Frontier Instrument Co., Chengdu, China). Additionally, the color indices *L* (indicative of lightness), *b* (representative of redness), and *a* (corresponding to yellowness) of the OPKEDFs and H-EWPGs were measured with an NH130 High-Quality Portable Color Difference Meter (Three-NH Colorimeter Co., Shenzhen, China), then the color difference (Δ*E* values) between OPKEDF (/H-EWPG) and the modified OPKEDFs (/H-EWPGs fortified with OPKEDFs) were calculated as follows:

(1) $$∆E=\sqrt{{\left(L-L_{0}\right)}^{2}+{\left(a-a_{0}\right)}^{2}+{\left(b-b_{0}\right)}^{2}}$$

where *a*_0_, *L*_0_ and *b*_0_ separately are the redness, lightness and yellowness of untreated OPKEDF.

### 3.7. Structural Investigations

#### 3.7.1. Microstructure Microscopy

First, OPKEDFs and H-EWPGs were coated with a 10 nm gold layer. Then, the samples were scanned using a scanning electron microscope (JOLE-JMS-5700E, Tokyo, Japan). The accelerating voltage, scale bar, and magnification were 10 kV, 1 μm, and 5000, respectively [17].

#### 3.7.2. Fourier-Transformed Infrared Spectroscopy

The OPKEDFs and H-EWGs were freeze-dried in a vacuum freeze-dryer (HFD-6, Heyuan Ice Equipment Co., LTD, Zhengzhou, China) and were then scanned using a Fourier-transform infrared (FT-IR) spectrometer (8400S, Shimadzu, Kyoto, Japan). PeakFit software v4.12 (Seasolve, Framingham, MA, USA) was employed to investigate the secondary structure of the H-EWPGs, following the procedure described by Bashash et al. [40], for wavenumbers ranging from 4000 to 400 cm^−1^.

### 3.8. Hydration Properties

The water-expansion ability (WEA), water-retention ability (WRA), and viscosity of the samples were determined by citing the same procedures from Zheng et al. [17], using a RAV-I5 viscometer (Shengyebao Niandu Instrument Factory, Guangzhou, China).

### 3.9. Characterization of Gels

#### 3.9.1. Water-Retention Ability

The centrifugation method was adopted to determine the water-retention ability (WRA) of the gel samples [28]. Briefly, approximately 2 g (*M*_0_) of H-EWGs was added to a centrifuge tube (50 mL), along with filter paper. After filtration at 4000× *g* at 4 °C for 20 min, the gel samples were weighed again (*M*_1_). The WRA (%) was calculated according to the following Equation:

(2) $$WRA\left(\%\right)=M_{1}/M_{0}\times 100\%\;$$

where *M*_0_ and *M*_1_ represent the gel weights before and after centrifugation, respectively.

#### 3.9.2. Optical Transparency

Each gel sample was transferred to a quartz colorimetric dish, and its absorbance was determined using a JH754PC UV-Vis spectrophotometer (Shanghai Jinghua Co., Ltd., Shanghai, China) at 600 nm [7].

### 3.10. Textural Properties of Gels

The textural properties of the H-EWGs, including their hardness, elasticity, cohesiveness, adhesiveness, chewability, and resilience, were tested using the TA Plus Texture Analyzer System (LLOYD Instrument Co., Hong Kong, China) with a P/36 R probe (cylinder with a diameter of 3.16 cm). The gels were placed in a cylindrical compression cell with a diameter of 4.4 cm, and the thickness of the gel was 2.2 cm. The cell was placed on the sample platform, and the test was started. The data were measured in TPA mode, and the test parameters are as follows: the trigger force, compression rate, and test, pre-test, and post-test speeds were 3 g, 50%, 1, 2, and 1 mm/s, respectively [9].

### 3.11. Statistical Analysis

Each determination was repeated three times. V.17.0 SPSS software (IBM Co., Chicago, IL, USA) was employed to analyze for significant differences in the data by means of analysis of variance (ANOVA, one-way) and Duncan’s multiple comparisons at a significance level of *p* < 0.05.

## 4. Conclusions

To improve the hydration properties and applications of OPKEDF in the food industry, enzymolysis combined with hydroxypropylation or crosslinking was used for the first time to modify OPKEDF. The results showed that enzymolysis in combination with hydroxypropylation or crosslinking remarkably improved the solubility and hydration properties of OPKEDF and increased its surface area. These composite modification processes enhanced the improving effect of OPKEDF in the gel and textural properties of EWPG, including its water-retention ability, pH, hardness, chewiness, and gumminess. OPKEDF-EPC had the highest SDF content, water-retention ability, and water expansion volume, and it showed the best effect in improving the hardness and chewiness of EWPG. Moreover, the addition of OPKEDF, OPKEDF-EH, and OPKEDF-EPC increased the random coil content of EWPG, densified its microstructure, and lowered its transparency (*p* < 0.05). 

Additionally, OPKEDF and OPKEDF-EH increased the EWPG’s resilience, but OPKEDF-EPC reduced it. Therefore, enzymolysis in combination with hydroxypropylation or crosslinking improved the hydration properties of OPKEDF, and the modified OPKEDFs enhanced the gel properties of EWPG. However, the underlying mechanisms by which these composite modification methods improve the effects of OPKEDF on the gel properties of EWPGs require further investigation. Further research is also needed to evaluate the effects of enzymatic enhancement and fiber modifications on the functional properties of food products beyond egg white protein gels.

## Figures and Tables

**Table 1:** Influences of different dual modifications on the chemical constitute, surface area, color, and hydration properties of oil palm kernel expeller dietary fiber.

Constituent	OPKEDF	OPKEDF-EH	OPKEDF-EPC
Moisture (g∙100 g^−1^)	6.75 ± 0.67 c	7.49 ± 0.27 c	8.35 ± 0.36 c
Fat (g∙100 g^−1^)	3.28 ± 0.21 c	2.54 ± 0.63 c	2.08 ± 0.27 c
Ash (g∙100 g^−1^)	3.19 ± 0.31 c	3.86 ± 0.16 c	4.05 ± 0.11 c
Protein (g∙100 g^−1^)	3.38 ± 0.28 c	3.39 ± 0.28 c	3.89 ± 0.39 c
Total dietary fiber (g∙100 g^−1^)	74.58 ± 5.56 c	72.37 ± 3.73 c	72.26 ± 3.57 c
Insoluble dietary fiber (g∙100 g^−1^)	70.61 ± 4.52 c	66.15 ± 3.33 d	64.51 ± 2.73 d
Soluble dietary fiber (g∙100 g^−1^)	2.97 ± 0.11 e	5.25 ± 1.09 d	7.79 ± 0.15 c
Cellulose (g∙100 g^−1^)	43.15 ± 2.98 a	27.9 ± 0.92 c	27.01 ± 3.04 c
Lignin (g∙100 g^−1^)	34.31 ± 1.99 a	22.85 ± 1.94 b	20.73 ± 2.23 b
Hemicellulose (g∙100 g^−1^)	21.07 ± 2.43 a	14.95 ± 0.54 b	13.47 ± 1.13 b
D_3,2_ (μm)	115.87 ± 2.58 c	79.68 ± 0.67 e	90.36 ± 2.47 d
Specific surface area (cm^2^/cm^3^)	1704.57± 7.85 e	2034.44 ± 9.87 c	1874.47 ± 12.53 d
*L*	42.7 ± 2.14 c	34.83 ± 2.02 d	30.25 ± 0.67 d
*a*	5.73 ± 0.23 d	8.79 ± 0.27 c	9.06 ± 0.44 c
*b*	8.62 ± 0.26 d	13.48 ± 0.36 c	14.91 ± 1.02 c
*ΔE*	Control	9.37	30.90
Water-retention ability (g/g)	2.34 ± 0.09 d	5.22 ± 0.18 c	4.91 ± 0.46 c
Water-expansion ability (mL/g)	0.92 ± 0.23 e	2.15 ± 0.17 d	3.43 ± 0.20 c
Viscosity (cP)	4.78 ± 0.27 e	7.71 ± 0.35 d	9.78 ± 1.65 c

OPKE—oil palm kernel expeller; OPKEDF—oil palm kernel expeller dietary fiber; OPKEDF-EH—oil palm kernel expeller dietary fiber modified by cellulase hydrolysis and hydroxypropylation; OPKEDF-EPC—oil palm kernel expeller dietary fiber treated with cellulase hydrolysis combined with phosphate crosslinking; TDF—total dietary fiber; SDF—soluble dietary fiber; IDF—insoluble dietary fiber. D_3,2_—the Sauter mean diameter of OPKEDFs. Different lowercase letters (c–e) in the same row indicate significant differences (*p* < 0.05). a and b represent the redness and yellowness of samples.

**Table 2:** Effects of different addition amounts of OPKEDFs on the texture properties of heat-induced egg white protein gel.

Gels	Amount (g/100 g)	Hardness (g)	Adhesiveness	Springiness	Gumminess	Chewiness (g)	Resilience
H-EWG	0	97.96 ± 7.50 e	−30.63 ± 2.17 b	0.77 ± 0.03 a	84.63 ± 3.65 e	78.65 ± 6.63 e	0.297 ± 0.012 d
H-EWG/OPKEDF	1	93.07 ± 5.08 e	−35.43 ± 1.57 c	0.82 ± 0.00 a	76.74 ±4.71 f	74.19 ± 5.69 ef	0.343 ± 0.007 b
	3	113.04 ± 6.19d e	−30.37 ± 1.44 b	0.83 ± 0.01 a	83.82 ± 1.49 e	84.36 ± 4.45 ef	0.347 ± 0.007 b
	5	124.49± 7.18 c	−26.22 ± 0.64 a	0.82 ± 0.00 a	104.00 ± 3.27 d	92.71 ± 3.36 c	0.318 ± 0.003 c
H-EWG/OPKEDF-EC	1	126.34 ± 11.38 c	−35.93 ± 3.27c	0.82 ± 0.02 a	106.51 ± 6.32 d	73.54 ± 3.98 e	0.335 ± 0.010 b
	3	119.88 ± 9.65 d	−47.74 ± 4.78 e	0.79 ± 0.03 a	113.22 ± 7.33 c	89.44± 1.05 cd	0.337 ± 0.007 b
	5	167.92 ± 3.95 b	−49.57 ± 4.39 e	0.83 ± 0.01 a	152.27 ± 7.69 a	102.23± 4.54 b	0.359 ± 0.003 a
H-EWG/OPKEDF-EPC	1	84.22 ± 7.27 f	−45.48 ±2.82 de	0.79 ± 0.01 a	88.56 ± 3.39 e	63.45 ± 2.44 f	0.232 ± 0.003 e
	3	174.91 ± 5.07 b	−43.36 ± 4.15 d	0.75 ± 0.03 a	119.16 ± 8.12 c	91.04 ± 4.06 c	0.195 ± 0.020 f
	5	195.00 ± 7.65 a	−51.36 ± 3.82 e	0.75 ± 0.00 a	140.72 ± 9.18 b	147.39 ± 2.63 a	0.152 ± 0.010 g

Different small letters (a–g) in the same column indicate a significant difference (*p* < 0.05).

## Data Availability

The original contributions presented in the study are included in the article, further inquiries can be directed to the corresponding author.
